# 5-hydroxyindolacetic acid (5-HIAA), a main metabolite of serotonin, is responsible for complete Freund's adjuvant-induced thermal hyperalgesia in mice

**DOI:** 10.1186/1744-8069-7-21

**Published:** 2011-03-30

**Authors:** Yong Chen, Florian Palm, Klaus-Peter Lesch, Manfred Gerlach, Rainald Moessner, Claudia Sommer

**Affiliations:** 1 Department of Neurology, University of Würzburg, Josef-Schneider-Strasse 11, Würzburg 97080, Germany; 2 Department of Psychiatry, Psychosomatics and Psychotherapy, University of Würzburg, Füchsleinstrasse 15, Würzburg 97080, Germany; 3 Department of Child and Adolescent Psychiatry, Psychosomatics and Psychotherapy, University of Würzburg, Füchsleinstrasse 15, Würzburg 97080, Germany; 4 Department of Psychiatry, University of Bonn, Sigmund-Freud-Strasse 25, Bonn 53105, Germany

## Abstract

**Background:**

The role of serotonin (5-hydroxytrptamine, 5-HT) in the modulation of pain has been widely studied. Previous work led to the hypothesis that 5-hydroxyindolacetic acid (5-HIAA), a main metabolite of serotonin, might by itself influence pain thresholds.

**Results:**

In the present study, we investigated the role of 5-HIAA in inflammatory pain induced by intraplantar injection of complete Freund's adjuvant (CFA) into the hind paw of mice. Wild-type mice were compared to mice deficient of the 5-HT transporter (5-HTT-/- mice) using behavioral tests for hyperalgesia and high-performance liquid chromatography (HPLC) to determine tissue levels of 5-HIAA. Wild-type mice reproducibly developed thermal hyperalgesia and paw edema for 5 days after CFA injection. 5-HTT-/- mice treated with CFA had reduced thermal hyperalgesia on day 1 after CFA injection and normal responses to heat thereafter. The 5-HIAA levels in spinal cord and sciatic nerve as measured with HPLC were lower in 5-HTT-/- mice than in wild-type mice after CFA injection. Pretreatment of wild-type mice with intraperitoneal injection of para-chlorophenylalanine (p-CPA), a serotonin synthesis inhibitor, resulted in depletion of the 5-HIAA content in spinal cord and sciatic nerve and decrease in thermal hyperalgesia in CFA injected mice. The application of exogenous 5-HIAA resulted in potentiation of thermal hyperalgesia induced by CFA in 5-HTT-/- mice and in wild-type mice pretreated with p-CPA, but not in wild-type mice without p-CPA pretreatment. Further, methysergide, a broad-spectrum serotonin receptor antagonist, had no effect on 5-HIAA-induced potentiation of thermal hyperalgesia in CFA-treated wild-type mice.

**Conclusion:**

Taken together, the present results suggest that 5-HIAA plays an important role in modulating peripheral thermal hyperalgesia in CFA induced inflammation, probably via a non-serotonin receptor mechanism.

## 

Serotonin (5-hydroxytryptamine, 5-HT) is present in serotonergic neurons in the CNS, and is released from platelets and mast cells during injury and inflammation in the periphery. 5-HT exerts algesic and analgesic effects in both the central and peripheral nervous systems depending on the site of action and on receptor subtype activation [1-8]. The 5-HT transporter (5-HTT), a member of the Na^+^/Cl^-^-dependent transporter family, plays a key role in central serotoninergic neurotransmission by controlling its intensity and duration through the reuptake of 5-HT that has been released from serotonergic terminals, somata and dendrites [9]. 5-HTT removes 5-HT from the synaptic cleft and determines the magnitude and duration of postsynaptic receptor-mediated signaling, thus playing a pivotal role in the fine-tuning of 5-HT neurotransmission [9,10]. In addition, 5-HTT is the target of antidepressants known as selective serotonin reuptake inhibitors (SSRIs) such as fluoxetine and paroxetine [11]. Mice with a genetic deficiency in 5-HTT (5-HTT-/-mice) have increased extracellular 5-HT levels [12], but the overall tissue content of 5-HT is reduced [13]. These changes make 5-HTT-/- mice an interesting model to study pain behavior. We recently observed that 5-HTT-/-mice, in contrast to wild-type mice, did not develop thermal hyperalgesia after chronic constriction injury (CCI) of the sciatic nerve [14].

In inflammation and after cell injury, 5-HT is released and converted to 5-hydroxyindolacetic acid (5-HIAA) by monoamine oxidase (MAO) and aldehyde dehydrogenase (ALDH). Cerebrospinal fluid (CSF) levels of 5-HIAA are used as indicators of serotonergic neuronal activity [15]. Despite a wealth of data about the 5-HT turnover rate and 5-HIAA concentrations in various tissues under normal and pathological conditions, an intrinsic role for 5-HIAA has not been shown, and 5-HIAA is regarded as an inactive metabolite.

To explore a possible active role of 5-HIAA in inflammatory pain, we measured pain-related behaviors and 5-HIAA levels in the sciatic nerve and spinal cord after hind paw inflammation-induced by complete Freund's adjuvant (CFA) in 5-HTT-/- mice and in wild-type mice. In addition, we investigated the effects of pretreatment with para-chlorophenylalanine (p-CPA), a 5-HT synthesis inhibitor [16] on pain behaviors and on 5-HIAA levels in wild-type mice after intraplantar CFA injection. Furthermore, the influence of exogenous 5-HIAA on CFA-induced thermal hyperalgesia was investigated in 5-HTT-/- mice and in wild-type mice.

## Materials and methods

### Animals

We used homozygous knock-out (5-HTT-/-) mice and littermate control wild-type mice (18-24 g) with a C57BL/6J genetic background, as described previously [13]. The animals were housed in a 14/10 h light/dark cycle with standard rodent chow and water available *ad libitum*. All experiments were approved by the Bavarian state authorities and performed in accordance with the European Communities Council Directive of November 24, 1986 (86/609/EEC) for the care and use of laboratory animals.

### Drugs and drug administration

Complete Freund's adjuvant (CFA) was purchased from Difco Laboratories (Detroit, USA), and para-chlorophenylalanine methyl ester hydrochloride (p-CPA), 5-hydroxyindolacetic acid (5-HIAA) and methysergide from Sigma-Aldrich (Munich, Germany). P-CPA, dissolved in normal saline (NS), was administrated by intraperitoneal (i.p.) injection at 300 mg/kg. 5-HIAA was dissolved in distilled water with 0.1% sodium metabisulfate as antioxidant. Injections of CFA (diluted 1:1 with PBS, 10 μl, 2 mg/ml), and 5-HIAA with an adjusted pH of 7.4 (5 μl, 4 μg/ml) were given subcutaneously into the plantar surface of one hind paw (i.pl.) with a Hamilton syringe coupled to a 30-gauge needle under light ether anesthesia. In the antagonist experiments, methysergide (5 μl, 2 mg/ml in NS) was administered i.pl. 5 min before 5-HIAA. Control mice received the same volume of respective vehicles (NS or distilled water with 0.1% sodium metabisulfate).

### Behavioral testing

Sensitivity to noxious heat was assessed using the device of Hargreaves et al. [17] purchased from Ugo Basile (Comerio, Italy). A radiant heat source was focused on the plantar surface of the hind paw; the latency from the initiation of the radiant heat until paw withdrawal (paw withdrawal latency, PWL) was measured automatically. A maximal cutoff of 20 s was used to prevent tissue damage. Each paw was tested three times and the mean withdrawal latency was calculated, with the exception of the experiments including 5-HIAA injection, where mice were tested only once. The interval between two trials on the same paw was at least 5 min.

Paw thickness was measured from the ventral to the dorsal paw surface with a micrometer gauge (resolution 0.1 mm). Care was taken to assure that the micrometer was placed at the same site on the paw for each measurement and at a similar location across all animals.

### Withdrawal latencies to heat and paw thickness of wild-type and 5-HTT-/- mice after CFA

**Figure 1:**
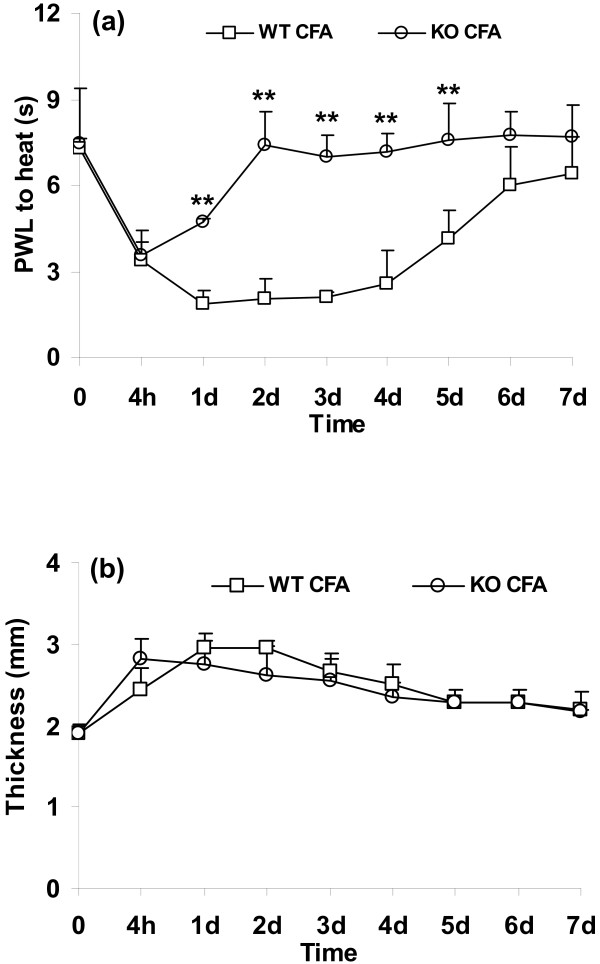
**Paw withdrawal latency (PWL) to heat (a) and paw thickness (b) at baseline and on distinct days after CFA in wild-type mice (WT) and 5-HTT-/- mice (KO)**. On the operated side, the reduction of PWL to heat was attenuated after CFA in 5-HTT-/- mice compared with wild-type mice (a, **p < 0.01 compared with WT CFA). Paw thickness increased after CFA without differences between genotypes (b). n = 4 for each group.

To reproduce the differences in pain behavior and paw thickness between wild-type and 5-HTT-/- mice after CFA as shown before [18], mice were injected with i.pl. CFA (WT CFA and KO CFA groups, n = 4 per group, Figure 1). For controls, NS was injected i.pl to both genotypes. PWL to heat and paw thickness were tested using the methods as described above.

### Influence of exogenous and endogenous 5-HIAA on CFA-induced thermal hyperalgesia

Two separate experimental designs were used to investigate whether 5-HIAA is involved in CFA induced thermal hyperalgesia, 1) Injection of 5-HIAA in 5-HTT-/- mice with known constitutive low levels of 5-HIAA, 2) Injection of 5-HIAA in wild-type mice after pharmacological reduction of 5-HT (and consequently, 5-HIAA) production by p-CPA pretreatment in wild-type mice.

**Figure 2:**
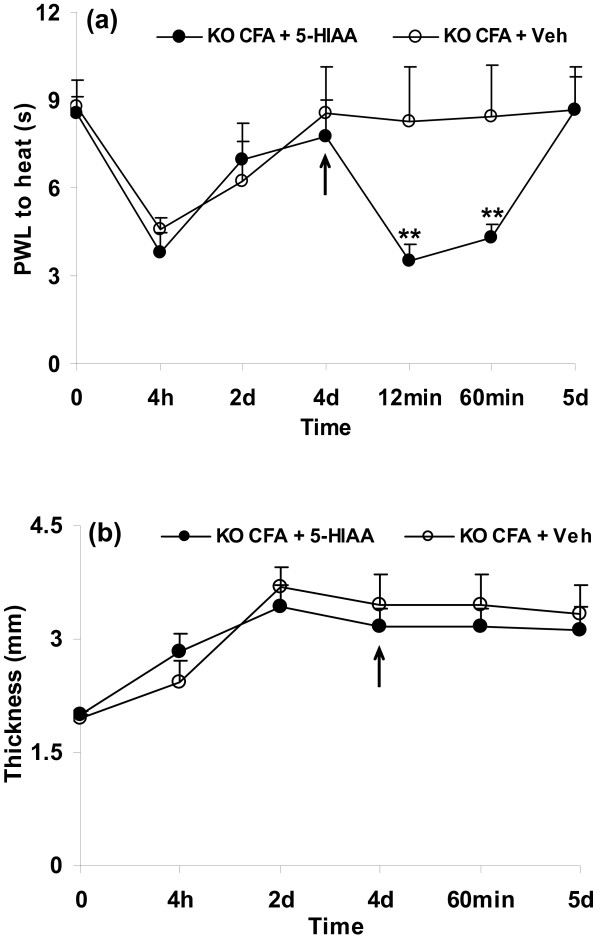
5-HIAA potentiated the reduction in paw withdrawal latency (PWL) to heat (a, **p < 0.01 compared to vehicle injection, a) but not paw edema (thickness, b) caused by CFA in 5-HTT-/- mice. Note: all animals used here were 5-HTT-/- mice. n = 8 for each group. Arrow: 5-HIAA or vehicle (water with 0.1% sodium metabisulfate) injections.

**Figure 3:**
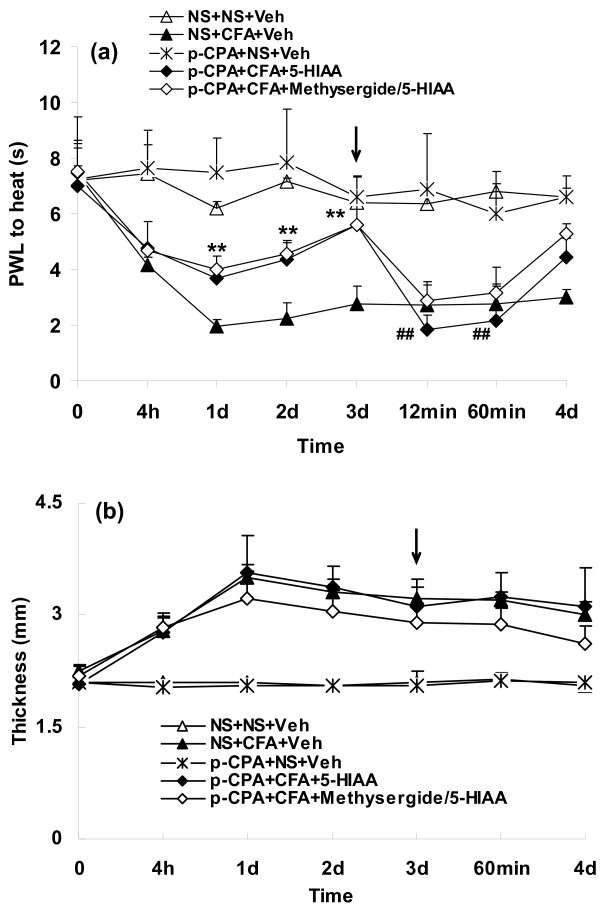
Pretreatment with p-CPA attenuated the CFA-induced reduction in paw withdrawal latencies (PWL)(a, **p < 0.01 compared with NS+ CFA treatment) but not paw edema (b). Subsequent injection of 5-HIAA increased CFA-induced thermal hyperalgesia of p-CPA pretreatment mice (a, ^##^*p *< 0.01 compared with values at day 3) but had no effect on paw edema (n.s., b). Furthermore, methysergide, a non-selective antagonist of 5-HT receptors, could not antagonize the effects of 5-HIAA. p-CPA itself had no significant effect on baseline PWL (a) and paw thickness (b). Note: all animals used here were wild-type mice. n = 4~5 for each group. Arrow: 5-HIAA or vehicle (water with 0.1% sodium metabisulfate) injections.

1) First, 16 5-HTT-/- mice were injected i.pl. with CFA. Mice were tested for the withdrawal latencies to heat and paw thickness for 4 consecutive days after the injection. On day 4, when withdrawal latencies were almost back to baseline, 8 mice were injected with i.pl. 5-HIAA and the other 8 mice received i.pl. injections of vehicle (KO CFA+5-HIAA and KO CFA+Veh groups, Figure 2). Mice were again tested after 5-HIAA or vehicle injections.

2) Eight wild-type mice were treated with 300 mg/kg p-CPA, a serotonin synthesis inhibitor, after assessing baseline pain thresholds and paw thickness. This treatment has previously been shown to reduce 5-HT levels by 60-80% in mice [19,20]. Pretreatment with p-CPA was followed by either i.pl. injection of CFA or NS (day 0, n = 4 per group), and post-drug thermal hyperalgesia and paw thickness were measured. On day 3 after these injections, mice were then injected either with i.pl. 5-HIAA or with i.pl. vehicle (p-CPA+CFA+5-HIAA and p-CPA+NS+Veh groups, Figure 3). As controls, two groups of wild-type mice without p-CPA pretreatment received CFA and vehicle (NS+CFA+Veh group, n = 4, Figure 3), or NS and vehicle (NS+NS+Veh group, n = 4, Figure 3). To identify whether the potentiation of thermal hyperalgesia induced by 5-HIAA was through serotonin receptors, methysergide, a broad-spectrum antagonist of 5-HT1, 5-HT2, 5-HT5, 5-HT6 and 5-HT7 receptors [21], was injected i.pl. 5 min before 5-HIAA injection on day 3 after CFA injection in wild-type mice pretreated with p-CPA (p-CPA+CFA+Methysergide/5-HIAA group, n = 5, Figure 3).

**Figure 4:**
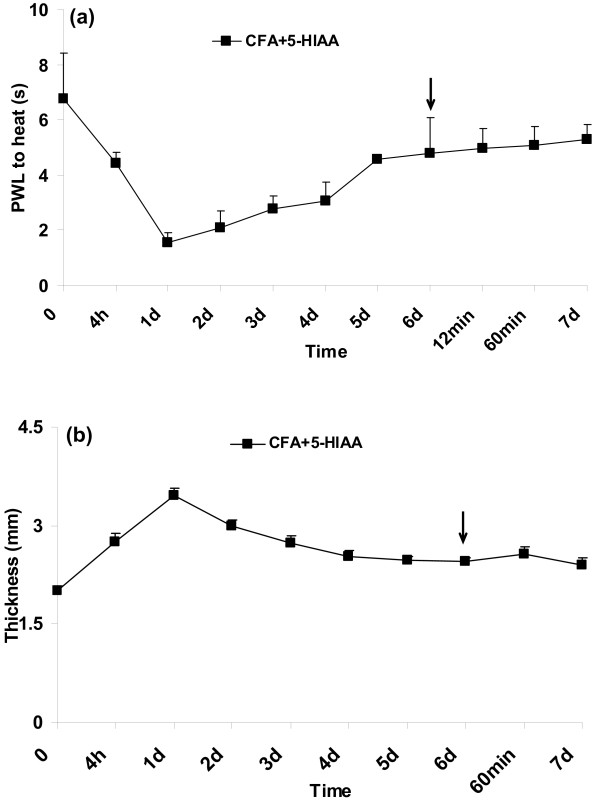
**5-HIAA had no effect on thermal hyperalgesia (a) and paw thickness (b) in wild-type CFA mice**. n = 4 for each group. Arrow: 5-HIAA injection.

**Figure 5:**
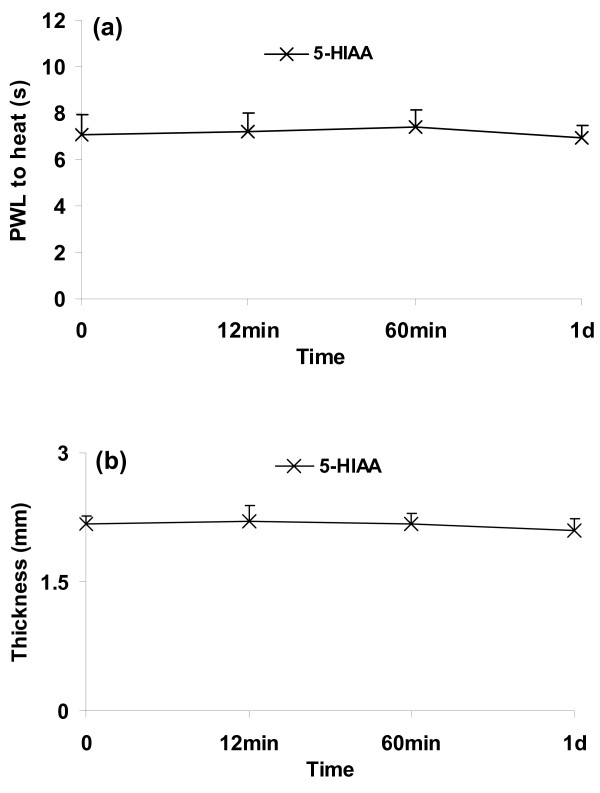
**5-HIAA *per se *had no effect on thermal hyperalgesia (a) and paw thickness (b) in wild-type naïve mice**. n = 4 for each group.

Additionally, one group of wild-type mice without p-CPA pretreatment received CFA, and on day 6, when pain thresholds were again close to baseline, 5-HIAA was i.pl. injected (CFA+5-HIAA group, n = 4, Figure 4). To investigate the effects of 5-HIAA *per se *on naïve wild-type animals, a separate group was injected with i.pl. 5-HIAA only (n = 4, Figure 5).

The experimental design is additionally summarized in Table 1.

**Table 1:** Experimental design and main behavioral results

Genotype	Treatment	Route	Effect
5-HTT +/+	CFA	i.pl.	Hyperalgesia
5-HTT -/-	CFA	i.pl.	Reduced hyperalgesia
5-HTT -/-	CFA+5-HIAA	i.pl.+i.pl.	5-HIAA restores CFA-induced hyperalgesia
5-HTT -/-	CFA+Veh	i.pl.+i.pl.	Vehicle does not affect CFA-induced hyperalgesia
5-HTT +/+	NS+NS+Veh	i.p.+i.pl.+i.pl.	No change
5-HTT +/+	NS+CFA+Veh	i.p.+i.pl.+i.pl.	Hyperalgesia
5-HTT +/+	p-CPA+NS+Veh	i.p.+i.pl.+i.pl.	No change
5-HTT +/+	p-CPA+CFA+5-HIAA	i.p.+i.pl.+i.pl.	CFA-induced hyperalgesia is attenuated by p-CPA pretreatment; 5-HIAA restores hyperalgesia
5-HTT +/+	p-CPA+CFA+Methysergide/5-HIAA	i.p.+i.pl.+i.pl./i.pl.	Methysergide does not antagonize 5-HIAA induced hyperalgesia
5-HTT +/+	CFA+5-HIAA	i.pl.+i.pl.	5-HIAA has no effect on CFA-induced hyperalgesia
5-HTT +/+	5-HIAA	i.pl.	No change

Note: Vehicle = water with 0.1% sodium metabisulfate

### Determination of 5-HIAA concentration

Tissue was collected from separate groups (n = 4 per group) of mice on day 2 and 7 after injection of CFA, of mice on day 3 after CFA or vehicle pretreatment with p-CPA or NS, and of control mice under deep barbiturate anesthesia. Samples were taken from the mid sciatic nerve with a length of 1 cm and from the L4/5 spinal cord (cut just proximally to the L4 and distally to the L5 spinal root). Samples were weighed and frozen at -80°C before further processing. For HPLC, as previously described [14], samples were sonicated under argon in ice-cold 150 mM H_3_PO_4 _and 500 μM diethylenetriamine pentaacetic acid and centrifuged at 35,000 × *g *for 20 min at 4°C. The supernatant was filtered through Millipore (Bedford, MA) Ultrafree-MC filter cups at 9000 × *g *for 1-2 h at 4°C. For the analysis of 5-HIAA and 5-HT, 50 μl portions of the supernatants were injected directly into an HPLC system with electrochemical detection (Gynkotek, Germering, Germany).

### Statistical analysis

Results are presented as means ± standard deviation. Differences between animal groups were studied for significance with one-way analysis of variance (ANOVA), which assessed the overall influence of genotype, injected side, time, and treatment after injections, followed by a post hoc general contrast comparison using Tukey's test. Significance was set at *p *< 0.05.

## Results

### Withdrawal latencies to heat and paw thickness of wild-type and 5-HTT-/- mice after CFA

Baseline values for paw withdrawal latency (PWL) to thermal stimuli and paw thickness did not differ between wild-type and 5-HTT-/- mice (Figure 1). Thermal hyperalgesia was observed on the CFA injected side in wild-type mice from day 1 until day 5 after CFA injection, and was maximal between days 1 and 3 (Figure 1a). Only moderate thermal hyperalgesia was observed on the CFA side of 5-HTT-/- mice on day 1, and none on the other test days (Figure 1a, **p < 0.01 compared to wild-type mice), reproducing previous findings with this genotype [18]. CFA injected paws were swollen from 4 h after the injection. There was no difference in paw swelling between 5-HTT-/- mice and wild-type mice (Figure 1b) The withdrawal latencies and paw thickness in saline injected mice of both genotypes did not change compared to baseline over the complete duration of the experiments (data not shown).

### Influence of exogenous and endogenous 5-HIAA on CFA-induced thermal hyperalgesia and paw edema in 5-HTT-/- and wild-type mice

On day 4 after i.pl. CFA injection, PWLs were almost back to baseline in 5-HTT-/- mice, whereas they were still significantly reduced in wild-type mice (Figure 1a). To determine whether the attenuation of thermal hyperalgesia in 5-HTT-/- mice might be causally connected to a depletion of endogenous 5-HIAA, mice were injected i.pl. with 5-HIAA or vehicle. Animals were again tested for the development of thermal hyperalgesia after 5-HIAA or vehicle injections. I.pl. injection of 5-HIAA on day 4 after i.pl. CFA induced an increase of thermal hyperalgesia 12 min to 60 min after injection (Figure 2a, **p < 0.01 compared with vehicle injections). CFA-induced paw swelling was not altered by either 5-HIAA or vehicle injections (Figure 2b).

**Figure 6:**
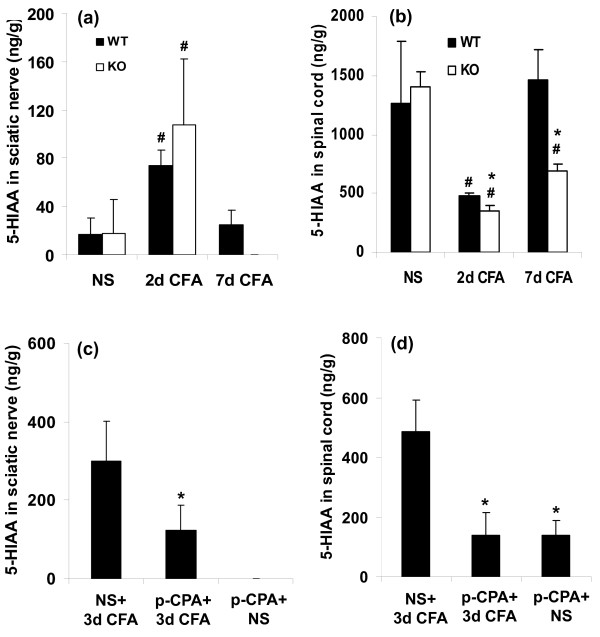
**5-HIAA measurements by HPLC from both genotypes on day 2 and 7 after CFA or saline in sciatic nerve (a) and spinal cord (b) of wild-type (black bars) and 5-HTT-/- mice (open bars)**. In control groups, there was no significant difference in 5-HIAA in sciatic nerve and spinal cord investigated between two genotypes. Two days and 7 days after CFA in 5-HTT-/- mice, the 5-HIAA was significantly reduced in sciatic nerve and spinal cord with the exception of 5-HIAA in sciatic nerve on day 2. (*p < 0.05 compared with wild-type mice after CFA). In sciatic nerve of 5-HTT-/- mice, 5-HIAA could not be detected on day 7 after CFA, and it was increased in both genotypes on day 2 after CFA. 5-HIAA was further reduced in spinal cord in both genotypes on day 2 after CFA, and in 5-HTT-/- mice on day 7 after CFA (^#^*p *< 0.05 compared with saline-treated groups respectively). On day 3 after i.pl. CFA or NS, 5-HIAA concentrations in sciatic nerve (c) and spinal cord (d) were decreased in wild-type mice pretreated with p-CPA (*p < 0.05 compared with pretreatment of NS group). n = 4~6 for each group.

To mimic the situation of reduced 5-HIAA that was present in 5-HTT-/- mice, wild-type mice were pretreated with i.p. p-CPA for 3 consecutive days to reduce endogenous 5-HT (~75% reduction in spinal cord, compared to NS treated mice) and 5-HIAA levels (see Figure 6). P-CPA itself did not affect baseline thermal pain thresholds and paw thickness (Figure 3a, b), but mice with p-CPA pretreatment had a significantly attenuated reduction in PWL (i.e. reduced thermal hyperalgesia) on day 1 to 3 after CFA injection (Figure 3a, **p < 0.01 compared to mice with NS pretreatment). Administration of 5-HIAA 3 days after CFA injection in mice with p-CPA pretreatment (i.e. 5-HIAA reconstitution) significantly increased thermal hyperalgesia (Figure 3a, **p < 0.01 compared with values at day 3) from 12 min to 60 min after the injection, but had no effect on paw swelling. Methysergide, a broad-spectrum antagonist of 5-HT receptors, did not antagonize the effects of 5-HIAA in thermal hyperalgesia (Figure 3a).

In an additional wild-type group without p-CPA pretreatment, 5-HIAA had no effect on CFA-induced thermal hyperalgesia (Figure 4a) and paw swelling (Figure 4b) in wild-type mice.

Furthermore, 5-HIAA *per se *had no effect on thermal pain thresholds and paw thickness of naïve wild-type mice (Figure 5).

### Tissue 5-HIAA concentrations

In saline treated control mice, 5-HIAA was detectable in both genotypes in sciatic nerve and spinal cord without significant differences between genotypes (Figure 6a and 6b). 5-HIAA levels were lower in spinal cord and sciatic nerve of 5-HTT-/- mice compared with wild-type ones at all time points examined after CFA (Figure 6a, b, *p < 0.05), with the exception of 5-HIAA in sciatic nerve on day 2 after CFA. Both genotypes had a significant increase in 5-HIAA levels in sciatic nerve but a significant decrease in spinal cord on day 2 after CFA compared with NS treatment (Figure 6a, b, ^#^*p *< 0.05). 5-HIAA was also reduced in spinal cord of 5-HTT-/- mice on day 7 after CFA (Figure 6b, ^#^*p *< 0.05). In p-CPA pretreated wild-type mice, 5-HIAA concentrations were decreased both in sciatic nerve and spinal cord 3 days after CFA compared with NS pretreated wild-type CFA mice (Figure 6c, d, *p < 0.05).

## Discussion

After a peripheral inflammation induced by CFA, thermal hyperalgesia in 5-HTT-/- mice was significantly reduced compared to wild-type mice, confirming previous findings [18]. Having observed strikingly low 5-HIAA levels in sciatic nerves of 5-HTT-/- mice after nerve injury [14], we here investigated whether 5-HIAA itself might be involved in the behavioral difference between genotypes. Several approaches were employed to explore the role of endogenous and exogenous 5-HIAA in CFA-induced thermal hyperalgesia. The results strongly suggest that 5-HIAA may not be an inactive metabolite but may itself be involved in the pathogenesis of thermal hyperalgesia caused by CFA.

We have previously shown that 5-HTT-/- mice do not develop thermal hyperalgesia after CCI or CFA, a model of neuropathic pain or inflammatory pain [14,18]. In mice with CFA, reduced 5-HT levels in the injured peripheral nerves correlated with diminished behavioral signs of thermal hyperalgesia [18]. Thus, in an inflammatory pain model, the reduced 5-HT content in 5-HTT-/- mice may be one possible mechanism by which 5-HTT-/- mice were protected from thermal hyperalgesia caused by CFA. However, 5-HT may not be the only candidate molecule to explain these findings. In the periphery, 5-HT is produced by enterochromaffin cells and transported into the tissues by platelets and mast cells. 5-HT is unable to penetrate the blood-brain and blood-nerve barrier, but a small amount of 5-HT is produced in neuronal cells and their terminals. In the inflammatory process and subsequent cell injury, 5-HT is released and converted to 5-HIAA by monoamine oxidase (MAO) and aldehyde dehydrogenase (ALDH). The 5-HTT is needed for uptake of 5-HT into the cells from the extracellular space. Thus, a functionally ablated 5-HTT in 5-HTT-/- mice entails reduced tissue 5-HT concentrations and a decreased content of 5-HIAA accordingly. Our HPLC results show that 5-HIAA levels in sciatic nerve 7 days after CFA, and in spinal cord 2 and 7 days after CFA, were significantly lower in 5-HTT-/- mice than in wild-type mice. Although thermal hyperalgesia was absent in 5-HTT-/- mice 2 days after CFA, there was no difference in 5-HIAA levels in sciatic nerve between two genotypes at this time point. It seems likely that the reduced thermal hyperalgesia in 5-HTT-/- mice is mainly connected to the reduced spinal 5-HIAA levels at this time point. It has been extensively demonstrated that descending serotonergic pathways, mainly derived from the rostroventral medulla (RVM), control spinal pain and produce either inhibitory or facilitatory effects depending on the receptor subtype activated [4,7]. Although it is still unknown if 5-HIAA shares the same receptors and has similar functions as 5-HT, the possible role of 5-HIAA in inflammatory pain at the supraspinal cord must not be neglected. Further experiments are needed to address. Collectively, we speculated that 5-HIAA might not be an inactive substance but have some intrinsic activity in this context. Our following results confirmed this point.

The first approach was to reconstitute 5-HIAA to 5-HTT-/- mice during the phase of recovery from CFA induced inflammation. In this setting, endogenous 5-HIAA was lower in sciatic nerves and spinal cords than in wild-type mice, and exogenous 5-HIAA injected into the inflamed paw notably enhanced thermal hyperalgesia in the knock-outs. This finding supports the assumption that 5-HIAA, like 5-HT, may participate in inflammatory pain in this model. 5-HIAA had no effect in naïve wild-type mice and in wild-type mice treated with CFA. This may indicate that in a mouse with normal or higher 5-HIAA levels, an additional application of exogenous 5-HIAA has no additional effect because all available receptors are saturated. Another explication for the finding of an increased sensitivity of 5-HTT-/- mice could be that these mice develop a sensitivity to 5-HIAA that wild-type mice do not have. Our second approach was to mimic the situation of reduced tissue 5-HIAA in wild-type mice. We therefore made use of pretreatment of wild-type mice with p-CPA, a competitive 5-HT biosynthesis inhibitor, which has been used as effective inhibitor of brain regional and spinal cord 5-HT and 5-HIAA synthesis [19,20,22-25]. P-CPA pretreatment significantly reduced CFA-induced thermal hyperalgesia, which was restored by reconstitution with exogenous 5-HIAA. Thus, also p-CPA pretreated wild-type mice, which have reduced endogenous 5-HIAA levels in spinal cord and sciatic nerve, develop sensitivity to exogenous 5-HIAA.

In contrast to the reduction of thermal pain induced by CFA in the 5-HTT-/- mice, the degree of paw swelling was not different between 5-HTT-/- mice and wild-type mice. In addition, pretreatment of p-CPA did not influence the paw edema induced by CFA. This is in accordance with previous data showing a lack of effect of 5-HT receptor antagonists on edema formation [26,27]. Inflammation is a complex, multifactorial process involving cell infiltration and release of multiple inflammatory mediators like cytokines, growth factors, neuropeptides, and serotonin [28-33]. It is thus likely that in the absence of 5-HT and 5-HIAA, other mediators are sufficient to induce paw swelling in mice. 5-HIAA did not alter CFA induced paw swelling neither in 5-HTT-/- mice nor wild-type mice with p-CPA pretreatment, and did not influence paw thickness of the wild-type naïve mice. It seems that 5-HIAA does not play an important role in inflammatory edema in these settings.

Some exogenous and endogenous compounds can produce one or more bioactive metabolites after degradation. For example, morphine-6-glucuronide (M6G), a major metabolite of morphine and a μ-opioid agonist, was reported to attenuate pain behavior in the hotplate test [34]. Norketamine, a metabolite of ketamine, produces analgesic effects during phase 2 of the formalin test [35]. Substance P (SP) N-terminus metabolite SP(1-7) can modulate formalin-induced pain [36]. It is interesting to notice that melatonin, a physiologically active derivative of 5-HT, plays an important role in pain modulation through its own receptors [37-39]. In addition, it has been shown that 5-HIAA *per *se can inhibit the activity of glutathione-s-transferase of pig brain [40]. There is as yet no information available about a receptor or pathway by which 5-HIAA could possibly exert its action. The effects of 5-HIAA-induced potentiation of thermal hyperalgesia were unaffected by methysergide, a broad-spectrum antagonist of 5-HT receptors [21], indicating that the function of 5-HIAA is not specifically mediated through the 5-HT receptors but probably a distinct way, although 5-HIAA shares similarities in its chemical structure with 5-HT. However, it is worthy of note that it cannot be fully excluded that 5-HIAA exerts its actions through 5-HT receptors because methysergide can only block 5-HT1, 5-HT2, 5-HT5, 5-HT6 and 5-HT7 receptor subtypes [21].

In conclusion, the present data provide evidence that the lack of thermal hyperalgesia in 5-HTT-/- mice after CFA administration is at least partially due to the lower 5-HIAA levels in nerve tissues of those mice. Furthermore, CFA-induced thermal hyperalgesia is markedly reduced in wild-type mice with p-CPA pretreatment, concordantly with the reduction in 5-HIAA content. 5-HIAA itself can potentiate CFA-induced thermal hyperalgesia in 5-HTT-/- mice and in wild-type mice with p-CPA pretreatment. Thus, our results directly and indirectly show an effect of 5-HIAA in CFA-induced thermal hyperalgesia in mice. This is, to our knowledge, the first report of an intrinsic role of 5-HIAA in pain modulation. This function of 5-HIAA will have to be considered in studies on actions of 5-HT in inflammation and on clinical dysfunction of the 5-HT system in pain.

## List of abbreviations used

5-HIAA: 5-hydroxyindolacetic acid; 5-HT: 5-hydroxytrptamine; 5-HTT: 5-HT transporter; CFA: complete Freund's adjuvant; HPLC: high-performance liquid chromatography; i.p.: intraperitoneal; i.pl.: intraplantar; p-CPA: para-chlorophenylalanine; PWL: paw withdrawal latency.

## Authors' contributions

YC and FP designed and carried out pain behavior studies, the data analyses, and drafted the manuscript. KPL, MG and RM contributed 5-HTT knockout mice, HPLC analyses and participated in manuscript editing. CS designed, coordinated and supervised the experiments, analyzed the data and wrote the manuscript. All authors read and approved the final manuscript. This publication was funded by the German Research Foundation (DFG) and the University of Wuerzburg in the funding programme Open Access Publishing.

## Competing interests

The authors declare that they have no competing interests.
